# The Impact of Whole-Body Electromyostimulation on Body Posture and Trunk Muscle Strength in Untrained Persons

**DOI:** 10.3389/fphys.2019.01020

**Published:** 2019-08-20

**Authors:** Oliver Ludwig, Joshua Berger, Stephan Becker, Wolfgang Kemmler, Michael Fröhlich

**Affiliations:** ^1^Department of Sports Science, Faculty of Social Sciences, University of Kaiserslautern, Kaiserslautern, Germany; ^2^Institute of Medical Physics, Friedrich-Alexander University Erlangen-Nürnberg, Erlangen, Germany

**Keywords:** WB-EMS, muscle training, trunk flexion, trunk extension, posture training

## Abstract

Muscular imbalances of the trunk muscles are held responsible for changes in body posture. At the same time, whole-body electromyostimulation (WB-EMS) has been established as a new training method that enables simultaneous stimulation of many muscle groups. This study was aiming to analyze if a 10 weeks WB-EMS training changes posture-relevant parameters and/or improves isometric strength of the trunk extensors and flexors, and if there are differences based on stimulation at 20 Hz and 85 Hz. Fifty eight untrained adult test persons were divided into three groups (control, CON; training with 20 Hz stimulation, TR20; training with 85 Hz, TR85). Anthropometric parameters, trunk extension and flexion forces and torques, and posture parameters were determined before (*n* = 58) and after (*n* = 53: CON: *n* = 15, TR20: *n* = 19, TR85: *n* = 19) a 10 weeks WB-EMS training program (15 applications, 9 exercises). Differences between the groups were calculated for pre- and post-tests using univariate ANOVA and between the test times using repeated (2 × 3) ANOVA. Comparisons of pairs were calculated *post hoc* based on Fisher (LSD). No differences between the groups were found for the posture parameters. The *post hoc* analysis of both trunk flexion and trunk extension forces and torques showed a significant difference between the groups TR85 and CON but no difference between the other group pairs. A 10 weeks whole-body electrostimulation training with a stimulation frequency of 85 Hz in contrast to training with a stimulation frequency of 20 Hz improves the trunk muscle strength of an untrained group but does not significantly change posture parameters.

## Introduction

Basically, body posture is based on the interaction of muscles, tendons, and bones. Changes of the habitual posture develop as the body adapts to routine daily postures (Harrison et al., 1999; Prins et al., 2008; Claus et al., 2009; Roussouly and Pinheiro-Franco, 2011; Drzal-Grabiec and Snela, 2012; Jung et al., 2016). It is assumed that permanent poor posture in daily routines leads to muscular and articular overload, which in turn results in physical problems (Bruno et al., 2012; Araujo et al., 2017; Jentzsch et al., 2017). Particularly posture issues such as hyperlordosis and a hunched back are considered to be the reasons for back problems (Jentzsch et al., 2017; Murray et al., 2017). Current studies show an interrelationship between individual posture parameters and the occurrence of lower back pain (Kim et al., 2006; Dolphens et al., 2012; Aggarwal et al., 2013).

Poor posture is currently assumed to be caused by muscular imbalances and dysfunctional body perception (Kim et al., 2006; Buchtelová et al., 2013). Muscular imbalances are understood to be an imbalance of strength between agonist and antagonist, which moves a joint’s resting position from its neutral position (Nadler et al., 2001; Frank et al., 2009; Buchtelová et al., 2013). The position of the pelvis in the sagittal plane is particularly important in this case (Schwab et al., 2006; Sorensen et al., 2015). Functionally, the pelvis is considered a seesaw that is kept in equilibrium by muscular activity (Buchtelová et al., 2013). The abdominal muscles (*Musculus rectus abdominis*, *Musculus transversus*), the gluteal muscles (*Musculus gluteus maximus*), and the hamstrings (*Musculus biceps femoris*, *Musculus semitendinosus*, *Musculus semimembranosus*) all seem to influence the position of the pelvis by lifting the anterior pelvic rim and thus reduce anterior pelvic tilt (Bridger et al., 1992; Nourbakhsh and Arab, 2002; López-Miñarro et al., 2012; Jeong et al., 2015). Preventive recommendations include strengthening the pelvis-straightening muscles (Kim et al., 2006; Ludwig et al., 2016). Physical therapy to correct poor posture starts with practicing targeted muscle activation and improving muscular strength, joint flexibility, as well as body perception (Pope et al., 1985; Calvo-Muñoz et al., 2012; Laird et al., 2014; Barczyk-Pawelec et al., 2015; Kim et al., 2015; Szczygiel et al., 2018).

A proven therapeutic option to strengthen muscles is neuromuscular electrical stimulation (NMES) (Doucet et al., 2012). This method is based on the application of electrodes to the skin, which generate an electric field that changes membrane potentials and thus results in muscle fiber contractions (Filipovic et al., 2012). Depending on the frequency of the voltage pulse applied, the efferent nerve is usually not involved (Gondin et al., 2005; Maffiuletti, 2010). Particularly in physical therapy, NMES can help to rebuild muscles atrophied due to an injury or after immobilization (Adams, 2018). In contrast to NMES, functional electromyostimulation (FEMS) means the combination of electrical stimulation with functional movements such as walking or lifting objects. Some studies have shown that FEMS is able to increase muscle strength (Coupaud et al., 2008), retard muscle atrophy (Gargiulo et al., 2011), and reduce pain (Koyuncu et al., 2010).

Whole-body electromyostimulation (WB-EMS) has been established as a new training method that enables simultaneous stimulation of many muscle groups, for example by means of electrode vests (Kemmler and von Stengel, 2013). In contrast to the electromyostimulation known from physical therapy, newer WB-EMS concepts promote the idea to perform active movements during stimulation, i.e., to add an active central-nervous muscle activation to the passive electric stimulation (Herrero et al., 2010; Amaro-Gahete et al., 2018). Therefore, the application of WB-EMS has to be grouped under FEMS, because movements are carried out at the same time as voltage is applied. Studies have shown that WB-EMS improves strength in elite soccer players (Filipovic et al., 2016) and in untrained middle-aged men (Kemmler et al., 2016b) by increasing the number of muscle fibers brought to contraction during an exercise movement (Kemmler et al., 2010).

A number of stimulation parameters, such as impulse amplitude, impulse type, and impulse frequency can be modified to control EMS training. Research is not in agreement in this context, though. Most working groups describe a frequency range between 20 and 110 Hz to stimulate as many muscle fibers as possible (Moreno-Aranda and Seireg, 1981; Binder-Macleod and Barrish, 1992; Jones, 1996; Dreibati et al., 2010; Weissenfels et al., 2018). Certain frequencies are said to elicit a stronger stimulation of specific muscle fibers (Dreibati et al., 2010). Stimulation frequencies of up to 50 Hz appear to activate mainly the slower type-I muscle fibers, while frequencies between 50 and 120 Hz seem to stimulate the faster type-II fibers. However, there is no scientific consensus (Kemmler and von Stengel, 2013; Weissenfels et al., 2018), and this means that studies examining the effect of WB-EMS on specific muscle groups must observe the presumable effects of different stimulation frequencies.

Studies on the influence of WB-EMS training on body posture do not exist to date. Considering the high prevalence of poor posture (Bansal et al., 2014) and the time-consuming physical therapy treatments required, the research demand concerning new intervention methods is substantial. Therefore, the question arises whether an unspecific WB-EMS training might contribute to an improvement of posture parameters. This could be the case if the neuromuscular balance of the trunk muscles connected to the pelvis could be changed by WB-EMS training. Strengthening the lumbar parts of the back extensor also appears to be useful from a therapeutic point of view because this is already a proven form of therapy to treat lower back pain (Kahanovitz et al., 1987; Mannion et al., 2001; França et al., 2010). It has already been shown that conventional EMS training is able to achieve an improvement of muscular strength of *Musculus erector spinae* to prevent lower back pain (Kahanovitz et al., 1987; Kemmler et al., 2017; Weissenfels et al., 2018). Based on the seesaw model of the pelvis WB-EMS training could, however, also have a negative effect on the pelvis position through unspecific strengthening of the lumbar back muscles by increasing the pelvic tilt and thus leading to a more pronounced lumbar lordosis. On the other hand, strengthening the thoracic sections of *M. erector spinae*, which acts as a trunk extensor, WB-EMS training could result in a reduced forward body tilt and reduced spinal column curvatures, thus having a posture-improving effect.

In the light of the above, any potential influence of WB-EMS training on posture-constituting muscle groups and thus on body posture in general is unclear. In gyms, WB-EMS training is usually performed unspecifically, i.e., many superficial muscle groups in trunk and gluteus are activated during a training session. General strengthening of the trunk muscles does not necessarily have to have an effect on body posture, because muscular relationships often seem to be more important than absolute muscle force (Buchtelová et al., 2013). For this reason and since this training method spreads very quickly, research has a very strong interest in delimiting potential preventive and curative effects from potentially posture-damaging effects. As WB-EMS training is less time-consuming compared to conventional strength training (usually, a complete whole-body workout only takes 20 min), it could become interesting for large target groups in both prevention and therapy.

This study is therefore aiming to analyze the following questions:

(1) Can a 10 weeks WB-EMS training change the posture parameters *flèche cervicale, flèche lombaire* and trunk anteversion?
(2) Does a 10 weeks WB-EMS training change the isometric strength of the trunk extensors and flexors?
(3) Is there a difference in the change of posture parameters *flèche cervicale, flèche lombaire* and trunk anteversion based on stimulation at 20 Hz and 85 Hz?

## Materials and Methods

### Test Persons

**FIGURE 1:**
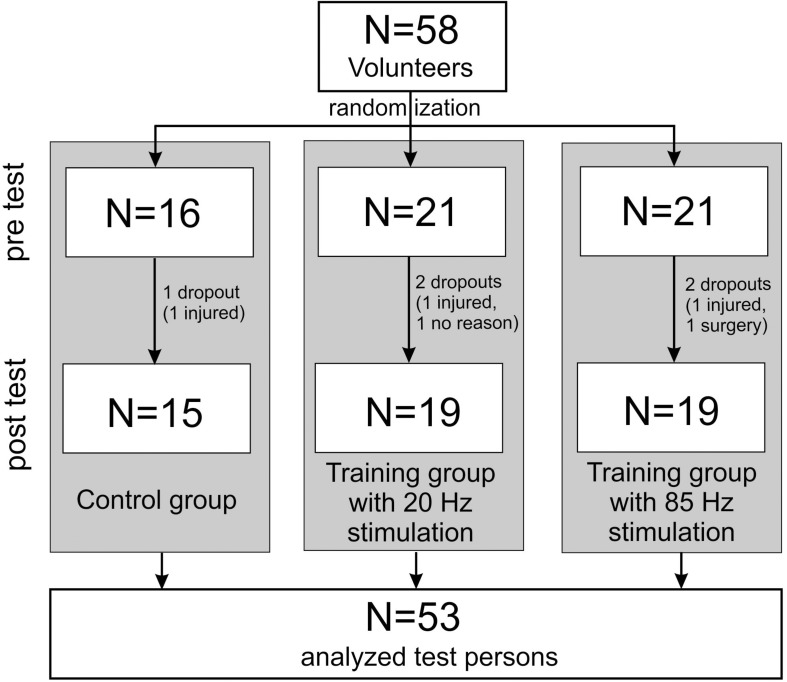
Flowchart according to the CONSORT recommendation (Schulz et al., 2010).

The sample size was calculated using G^∗^Power 3.1 (University of Kiel, Germany). For a repeated (2 × 3) ANOVA (within and between interactions, *f* = 0.3, α = 0.05) a minimum group size of 48 persons was calculated (power 0.958), which we increased due to the expected drop outs. Finally, 58 test persons participated in the study. They were recruited by means of flyers distributed on the university campus. Five test persons did not complete the 10 weeks training and were removed from the study. In the end, the data from 53 test persons was included in the evaluation (see Table 1 and flowchart Figure 1). All participating test persons did not have any previous experience or knowledge concerning EMS training. The test persons had to be between 18 and 40 years old, were not to perform any regular athletic activity, and needed to be free of internal and orthopedic limitations. Muscle stretchability and joint mobility were not explicitly examined before the start of the study. However, since all participants were able to perform all movements in the required range of motion (ROM) during the dynamic exercises, we assume that there was no movement restriction relevant for our study. All test persons were informed about the process and objectives of the study and gave their written consent before the study started. In order to exclude any risks associated with EMS training, a comprehensive anamnesis questionnaire had to be completed (Kemmler et al., 2016a). The study was approved by the ethics commission of the Technical University Kaiserslautern (ref. no. 02/17) and was conducted based on the Declaration of Helsinki (World Medical Association [WMA],, 2013).

**TABLE 1:** Anthropometric data of the three groups (means ± standard deviation).

	**CON**	**TR20**	**TR85**
N (total/men/women)	15/4/11	19/8/11	19/10/9
Age [years]	25.60 ± 2.80	24.84 ± 3.82	24.50 ± 4.40
Height [m]	168.22 ± 7.07	174.26 ± 7.87	176.72 ± 9.78
Weight [kg]	67.06 ± 19.93	74.04 ± 16.26	73.32 ± 15.14

### Measurements

**FIGURE 2:**
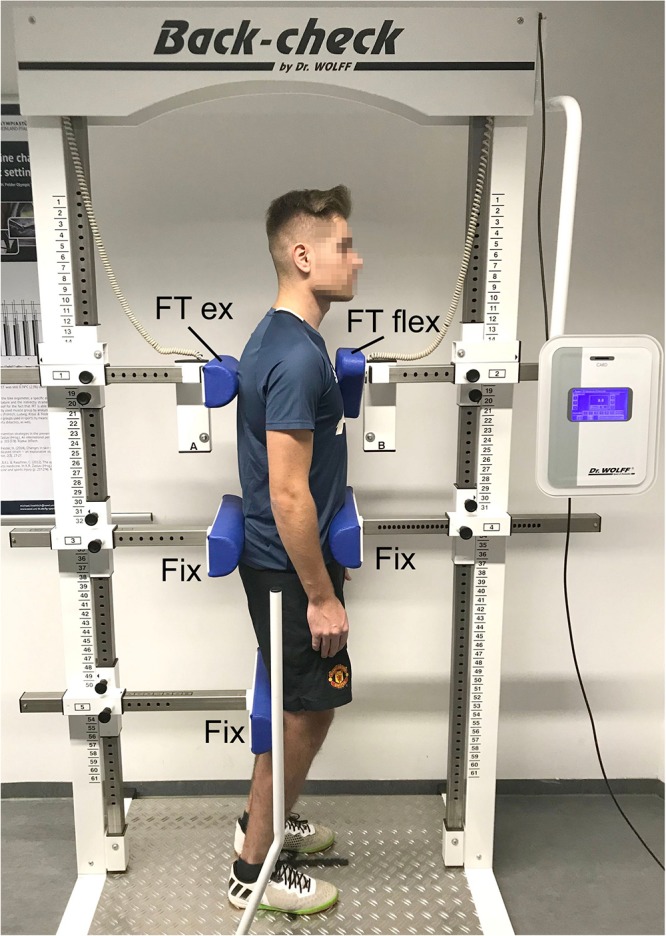
Placement and fixation of a test person in the isometric force measurement device. FT ext, force transducer extension; FT flex, force transducer flexion; Fix, fixation pads.

In both the initial and the final examination, the following anthropometric parameters were determined: body weight, height, and body fat percentage. The static trunk extension and flexion forces were measured by means of the isometric force testing device Back Check 607 (Dr. Wolff GmbH, Arnsberg, Germany). This required the test persons to stand with their arms hanging loosely and with slightly bent knee joints, fixated in the sagittal plane at the iliac crest area by one dorsal and one ventral pad. The flexion in the knee joints served to reduce the influence of the iliopsoas, since we only wanted to measure the strength of the trunk muscles. Two pads with force transducers were placed without pressure at the sternum and between the shoulder blades at an individual height (Figure 2). Alternatingly, three maximum strength measurements of the trunk flexion and the trunk extension were performed for 5 s each and 30 s breaks in between the measurements. The test persons were instructed to press against the pads as strongly as they could. If the isometric strength value of the last measurement was the largest, the sequence was continued after 30 s breaks until the value decreased. The highest values for extension and flexion were included in the evaluation. The flexion and extension torques were calculated as M = F^∗^(vertical distance between force transducer pad and pelvis fixation pad).

The reliability of this measurement system was analyzed in other studies and found to be in a good range [intraclass correlation coefficient 0.76–0.89 (Scheuer and Friedrich, 2010)].

**FIGURE 3:**
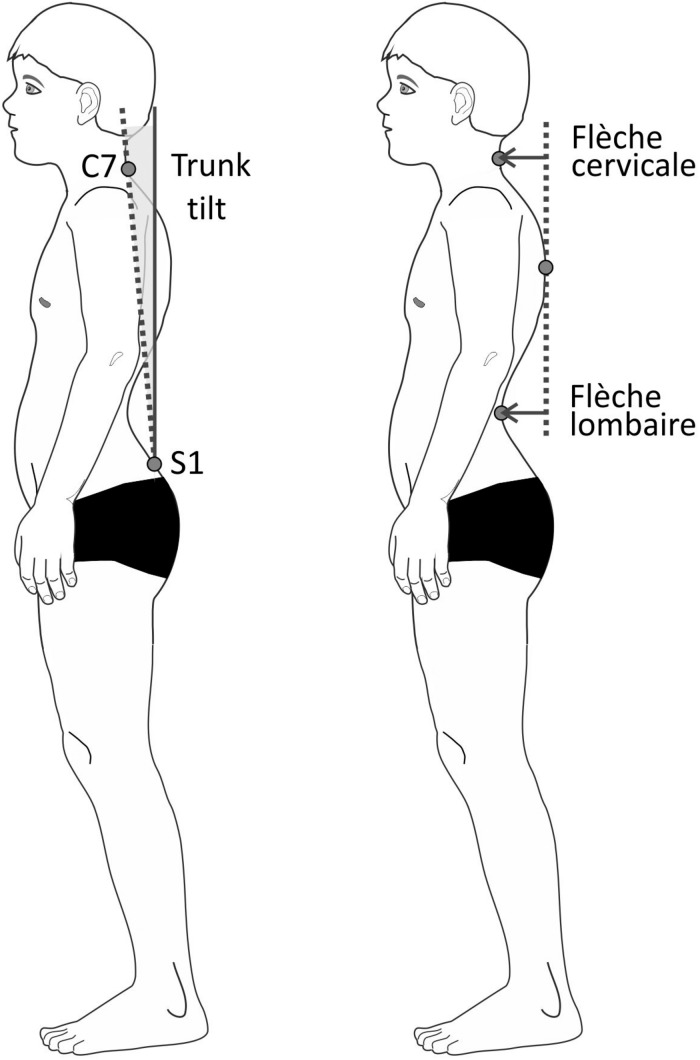
Schematic drawing of the posture parameters trunk tilt (left) and flèche cervicale and lombaire (right).

To evaluate posture, surface scans of the entire body were performed using the Paromed 4D Sanner (Paromed GmbH, Neubeuern, Germany). For those scans, the test persons stood barefoot and shirtless or wearing a sports bra about 2.5 m away from the scanner. They were instructed to stand at ease (habitual posture), let their arms hang loosely, look straight ahead, and breathe normally. Anatomic landmarks such as C7, S1, and the PSIS (posterior superior iliac spines) were marked by means of adhesive stickers on the skin (Patias et al., 2010). The PSIS markers were not used for further calculations but are required by the software used. By projecting a coded light stripe grid on to the body, the system reconstructed the three-dimensional contours of the body’s back. Three scans were averaged. The total measurement itself took about 10 s. The following parameters were calculated based on the 3D representation (Figure 3):

(1) *Flèche cervicale*: horizontal distance between the point of strongest thoracic kyphosis and the point of the lowest neck lordosis in the sagittal plane.
(2) *Flèche lombaire*: horizontal distance between the point of strongest thoracic kyphosis and the point of the lowest lumbar lordosis in the sagittal plane.
(3) Trunk anteversion: angle between the connecting line C7-S1 against the vertical axis in the sagittal plane.

*Flèche cervicale* and *flèche lombaire* are orthopedic measurements that show the depth of the curvature of the spine by using the distance of the extreme points of the lordosis and the thoracic kyphosis. Increased values suggest posture deficits (Stagnara et al., 1982; Vrtovec et al., 2009; Allier and Monnet, 2013). Both parameters were additionally calculated as percentage values by having been relativized to the vertical distance between C7 and S1.

### Training

The participating test persons were randomly assigned to two training groups (TR20, TR85) and the control group (CON) by lots. At no time during the study were the members of the training groups or the investigators informed about this assignment (training groups were double-blinded).

The CON did not perform any athletic activity during the 10 weeks training phase. In all, the training groups performed a total of 15 WB-EMS training sessions. They alternatingly exercised once or twice a week, so that an average of 1.5 training sessions was performed per week (Kemmler et al., 2016b, 2018). Before the training phase started, all test persons took part in a familiarization session in order to get used to the imminent WB-EMS application (Jee, 2018). The familiarization session took 12 min and was characterized by reduced voltage intensity to avoid muscular overload (Kemmler et al., 2016a). At the same time, the test persons were taught the proper exercise techniques and learned about the RPE (rating of perceived exertion) scale [0 = no exertion, 10 = maximum exertion (Borg and Kaijser, 2006)].

Stimulation was applied in line with common parameters: impulse width of 350 μs, bipolar impulse without impulse increase, 4 s load and 4 s break intervals, overall training duration 20 min (Kemmler et al., 2016a). Both training groups differed only in the stimulation frequency applied, i.e., 20 Hz (TR20) and 85 Hz (TR85), respectively. The test persons were instructed to exercise with an intensity of 6 (“hard”) on the modified Borg scale, while maintaining the entire ROM during the exercise. In order to ensure adequate exertion, the intensity of the electric impulses both during the sessions and between the sessions was adapted by the investigator. Every 2–3 min, the participants provided feedback and the intensity for each muscle group was changed in a way that the participant felt the exertion to be “hard”. The devices produce a maximal peak output voltage of 75 V at 1 kOhm (corresponding to a current of 75 mA), but do not display the actual intensity in absolute voltage. The output is only displayed in device-specific units from 0–100, in proportion to the initial voltage. The intensity data was noted at the end of each training session for each muscle group.

The training times were kept at a constant level for each test person in order to avoid influences caused by the time of day. Before each training session, a new anamnesis questionnaire on the current condition was completed. This was to exclude potential contraindications (e.g., dizziness, nausea, and pain) and to ensure safe training for the test persons. The test persons were instructed to drink sufficient liquids before the training started (at least 500 ml). During the course of the study, the test persons were instructed not to perform any additional physical training. This was asked repeatedly at the beginning of each training session. They were also requested not to physically exert themselves in the preceding 24 h by everyday activities. Furthermore, the questionnaire was used to make sure that medication or pain killers had not been taken, so that overexertion due to a missing ability to assess the extent of exertion was avoided.

### Devices and Exercises

**FIGURE 4:**
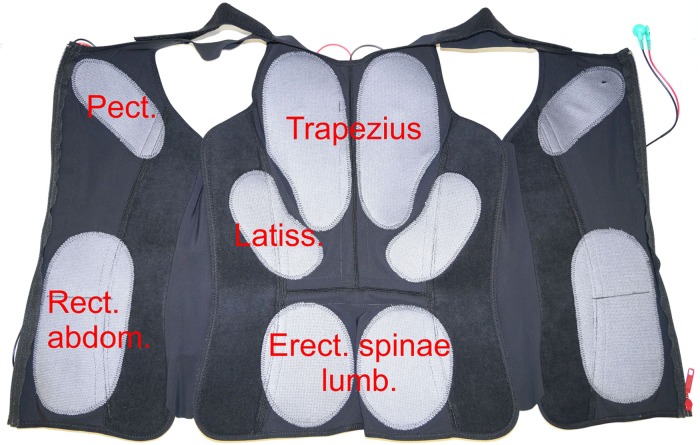
Positioning of the electrodes in a typical training vest. Only the largest target muscles below the electrodes are mentioned. Pect. – *M. pectoralis*, Trapezius – *M. trapezius*, Latiss. – *M. latissimus dorsi*, Rect. abdom. – *M. rectus abdominis*, Erect. spinae lumb. – *M. erector spinae* pars lumbalis.

A Miha Bodytec 2 WB-EMS (Miha Bodytec, Augsburg, Germany) was used. This system consists of an impulse transmitter with control panel and optical feedback. A 2 m cable connects the training vests including the integrated electrodes to the system. The training vests were available in various sizes, which were tightly attached to the upper body. The cranial tip of the sternum served as an anatomical reference for the correct placement of the vest. Caudal, the vest reached up to the iliac crests. The electrode surfaces and arrangement of all vests were proportional in all sizes (Figure 4). Upper arms and thighs were also equipped with additional circular electrodes that were attached with velcro at the level of the muscle bellies. The gluteus was stimulated by flat electrodes integrated into a belt that was closed at the abdomen. All electrodes and accessories were provided by the same manufacturer (Miha Bodytec, Augsburg, Germany). The system allows free programming of the stimulation parameters, whereas the stimulation parameters, except the applied voltage, are the same for all electrodes. The device settings were saved on a chip card for each test person.

Exercises were selected based on the exercises integrated in the WB-EMS device used. They were therefore representative of a typical exercise program performed in a gym, targeting as many stimulated muscle groups as possible. These exercises were performed:

(1) Dynamic squats (15 repetitions).
(2) Dynamic trunk flexion with retracted arms (12 repetitions).
(3) Static knee presses against own resistance (12 repetitions).
(4) Dynamic side lunge, left and right (10 repetitions each).
(5) Dynamic crunches, diagonal, left and right (10 repetitions each).
(6) Dynamic squats, wide stand (15 repetitions).
(7) Dynamic one-leg stand with lifting one leg, elbow to contralateral knee, left and right (10 repetitions each).
(8) Dynamic overextension of the trunk (12 repetitions).
(9) Static forward lunge, left and right (12 repetitions).

Active movements were initiated just before the electrical impulse was applied and continued to be performed during the entire impulse duration. Between impulses, the participants adopted a resting position.

The training was conducted exclusively under supervision of trained investigators with a support ratio of 1:2. Both safety and accurate exercising were ensured by the permanent supervision by the investigator (Moreno-Aranda and Seireg, 1981).

### Statistics

Differences between the groups (TR20, training at 20 Hz stimulation; TR85, identical training at 85 Hz stimulation; CON, untrained control group) were calculated for pre- and post-tests using univariate ANOVA (XLSTAT 2018.4, Addinsoft, Paris, France). Group differences between the test times were calculated using repeated (2 × 3) ANOVA. Comparisons of pairs were calculated *post hoc* based on Fisher (LSD). A possible influence of sex on the percentual increase of flexion and extension torques was calculated using (2 × 3) ANOVA. The significance level was set at 5%.

## Results

A significant difference between the three groups was not found for any of the posture parameters measured, neither at the beginning nor at the end of the study. Only the upper body anteversion decreased over time, but for all groups including the control group with no significant differences between the groups (TR85 vs. CON: *p* = 0.14, TR85 vs. TR20: *p* = 0.96; TR20 vs. CON: *p* = 0.16). All other posture parameters did not change. Table 2 shows the results of the statistic tests.

**TABLE 2:** Temporal development (pre = base line, post = after 10 weeks) of the posture parameters. CON, control group; TR20/TR85, training groups with stimulation frequencies of 20 Hz/85 Hz. The lower rows show the results of the repeated (2 × 3) ANOVAs.

	**Trunk tilt pre^#^**	**Trunk tilt post^#^**	**Flèche cervicale pre**	**Flèche cervicale post**	**Flèche cerv. % pre**	**Flèche cerv. % post**	**Flèche lombaire pre**	**Flèche lombaire post**	**Flèche lombaire % pre**	**Flèche lombaire % post**
CON	−3.47 ± 2.59	−3.17 ± 1.66	6.44 ± 1.76	6.13 ± 1.40	13.86% ± 3.56	13.15% ± 2.48	4.43 ± 1.16	3.96 ± 0.78	9.52% ± 2.39	8.54% ± 1.65
TR20	−3.19 ± 2.57	−2.29 ± 1.34	5.98 ± 1.59	5.45 ± 1.28	12.32% ± 3.21	11.19% ± 2.45	3.48 ± 1.71	3.22 ± 1.51	7.15% ± 3.50	6.63% ± 3.13
TR85	−3.67 ± 2.08	−2.55 ± 1.68	6.33 ± 1.73	6.12 ± 1.72	12.96% ± 3.05	12.50% ± 2.89	3.85 ± 1.10	3.96 ± 1.01	7.97% ± 2.38	8.14% ± 2.03
ANOVA (time)	df = 1; *F* = 6.56		df = 1; *F* = 3.00		df = 1; *F* = 3.30		df = 1; *F* = 2.20		df = 1; *F* = 2.40	
	*p* = **0.01^∗^**		*p* = 0.09		*p* = 0.07		*p* = 0.14		*p* = 0.12	
ANOVA (group^∗^ time)	df = 2; *F* = 1.14		df = 2; *F* = 0.23		df = 2; *F* = 0.23		df = 2; *F* = 1.51		df = 2; *F* = 1.44	
	*p* = 0.33		*p* = 0.79		*p* = 0.79		*p* = 0.23		*p* = 0.24	

*^#^Trunk tilt: negative values indicate anterior upper body tilt. Significant differences at *p* = 0.05 are marked in bold and with ^∗^.*

The **trunk flexion force** values were not significantly different between the groups at the beginning of the study (*df* = 2, *F* = 1.44, *p* = 0.25). At the end of the study, significant differences were identified for the factor *time* (*df* = 1, *F* = 36.97, *p* < 0.0001) and for the interaction *group^∗^time* (*df* = 2, *F* = 3.92, *p* = 0.02). The *post hoc* analysis showed a significant difference between the groups TR85 and CON (*p* = 0.02). There was no difference between the other group pairs (TR85 vs. TR20: *p* = 0.31; TR20 vs. CON: *p* = 0.16).

The **trunk flexion torque** values showed comparable results: they were not significantly different between the groups at the beginning of the study (*df* = 2, *F* = 1.61, *p* = 0.21). At the end of the study, we found significant differences for the factor *time* (*df* = 1, *F* = 31.90, *p* < 0.0001) and for the interaction *group^∗^time* (*df* = 2, *F* = 3.45, *p* = 0.04). The *post hoc* analysis showed a significant difference between the groups TR85 and CON (*p* = 0.03), but no difference between the other group pairs (TR85 vs. TR20: *p* = 0.42; TR20 vs. CON: *p* = 0.14).

There were also no group differences at the beginning of the study for the **trunk extension force** (*df* = 2, *F* = 1.38, *p* = 0.26). After the treatment, significant effects were identified for the factor *time* (*df* = 1, *F* = 56.59, *p* < 0.0001) and for the interaction *group^∗^time* (*df* = 2, *F* = 4.27, *p* = 0.02). The *post hoc* analysis showed a significant difference between the groups TR85 and CON (*p* = 0.04). There was no difference between the other group pairs (TR85 vs. TR20: *p* = 0.49; TR20 vs. CON: *p* = 0.15).

For the **trunk extension torque** we could not find a significant difference between the groups at the beginning of the study (*df* = 2, *F* = 1.46, *p* = 0.24). At the end of the study, significant differences for the factor *time* (*df* = 1, *F* = 44.84, *p* < 0.0001) and for the interaction *group^∗^time* (*df* = 2, *F* = 4.37, *p* = 0.02) could be found. The Fisher *post hoc* analysis showed a significant difference between the groups TR85 and CON (*p* = 0.04), and no difference between the other group pairs (TR85 vs. TR20: *p* = 0.55; TR20 vs. CON: *p* = 0.13).

The percentage improvement in the training groups did not differ between men and women (extension torque increase in %: men: 20.18% ± 16.64 vs. women 19.2% ± 17.5; flexion torque increase in %: men: 18.1% ± 12.03 vs. women 16.2% ± 15.9). A 2 × 3 ANOVA (sex, group) did not provide significant results; based on the Type III sum of squares, the variable sex did not bring any additional significant information (flexion: *df* = 1, *F* = 0.20, *p* = 0.66, extension: *df* = 1, *F* = 0.06, *p* = 0.80).

Body mass index did not change significantly during the study (*group^∗^time*: *F*(2) = 0.68, *p* = 0.52). This applied to all groups.

## Discussion

The objective of this study was to find out the extent to which unspecific WB-EMS training over a period of 10 weeks would achieve an improved body posture and increased trunk muscle strength in untrained persons, and whether any differences exist in terms of stimulation frequency.

### Trunk Muscle Strength

In the training group stimulated with 85 Hz, we were able to prove a significant improvement of the isometric strength and torque of trunk extension and flexion compared to the control group. The strength increases identified were on average between 15.0 and 21.4% (force) and between 15.9 and 26.6% (torque) for the groups in training (Table 2) and were of a similar size as increases found in other studies (Kemmler et al., 2016b, 2018). In the WB-EMS training, large electrodes in the areas of *M. rectus abdominis* (flexion), the lumbar and thoracic *M. erector spinae*, and the *Mm. multifidii* (flexion) induced contractions, so that an adequate training stimulation and corresponding muscle strengthening over the 10 weeks of the study can be assumed (Ng and Richardson, 1994). However, we also found force increases between 4.8 and 7.4% and torque increases between 4.4 and 9.2% in the control group. We interpret this finding as test habituation, i.e., that familiarity with the task of isometric maximal strength exercises was higher in the post-test and had a positive effect on the initially unfamiliar isometric strength development.

No significant difference was identified in the group stimulated at 20 Hz (Table 3). This difference between the groups was unexpected because the back musculature (*M. erector spinae*), which is important for posture control, consists of mainly slow type-I fibers [men 62.0 ± 9.3%, women 67.8 ± 10.5% (Mannion et al., 2001)]. We had actually expected a higher strength increase in the TR20 group, though, because the lower stimulation frequency presumably stimulates rather the slow muscle fibers (Cabric et al., 1988; Sillen et al., 2013; Kemmler et al., 2016b). The straight abdominal muscles, on the other hand, consist of a slightly higher percentage of type-II fibers [type I 46.1%, type II 53.9% (Johnson et al., 1973)] and should therefore be more susceptible to an 85 Hz stimulation. However, Gregory and Bickel found that an EMS-induced recruitment of motor units does not always proceed selectively. Type I and type II fibers seem to be recruited by EMS without adhering to any specific sequence (Gregory and Bickel, 2005). We therefore assume that muscle fibers were activated independently of the stimulation frequency, and that this fiber activation did not primarily depend on the fiber type, but rather on the physical location of the electrodes. Since the tetanic contraction of muscle fibers increases from a stimulation frequency of 20 Hz and more (Smith et al., 1985), we assume that the applied training stimulus was too low in the group training at 20 Hz. In addition, the duty cycle (on-off ratio) of the muscle fibers was more than four times lower with the lower stimulation frequency than with the 85 HZ stimulation. However, the duty cycle seems to play an important role in generating a training stimulus (Lloyd et al., 1986). In a comprehensive review, Filipovic et al. (2012) recommend a stimulation frequency greater than 50 Hz in order to generate a stimulation intensity that is sufficient to activate strength adaptation. Collins et al. (2007) were additionally able to prove that higher EMS stimulation frequencies (50–100 Hz) lead to electrically evoked sensory potentials, which induce the spinal motor neurons via reflex circuits to activate additional motor units. This contribution to muscle contraction, which is generated by the central nervous system, does not develop with frequencies < = 20 Hz and seems to max out at frequencies > = 80 Hz (Dean et al., 2007). Dean et al. (2007) were able to achieve an additional strength increase of 10.2% of the maximum voluntary contraction by applying four two-second stimuli. For this study, the stimulation was applied in 4 s stimuli, i.e., they were similar in intensity. We therefore assume that the lack of additional central-nervous contractions at an applied frequency of 20 Hz resulted in a lower training stimulus and thus in lower strength increases.

**TABLE 3:** Temporal development (pre = base line, post = after 10 weeks) of the trunk extensor (Ext) and flexor (Flex) forces and torques, the percentual improvement (Delta), and the ratio “extension/flexion” (Ext/Flex). CON, control group; TR20/TR85, training groups with stimulation frequencies of 20 Hz/85 Hz.

	**Force Flex pre [N]**	**Force Flex post [N]**	**Force Delta [%]**	**Torque Flex pre [Nm]**	**Torque Flex post [Nm]**	**Torque Delta Flex [%]**	**Ext pre [N]**	**Ext post [N]**	**Delta [%]**	**Torque Ext pre [Nm]**	**Torque Ext post [Nm]**	**Torque Delta Ext [%]**	**Ext/Flex pre [-]**	**Ext/Flex post [-]**
CON	419.2 ± 173.4	439.2 ± 196.2	4.8%	12.6 ± 7.5	13.2 ± 8.3	4.4 ± 12.7	558.5 ± 206.5	599.6 ± 205.0	7.4%	16.9 ± 9.0	18.0 ± 9.0	9.2 ± 12.5	1.4	1.4
TR20	478.6 ± 175.7	550.3 ± 197.5	15.0%	15.7 ± 7.6	18.1 ± 9.2	15.9 ± 10.8	590.3 ± 180.4	716.3 ± 175.3	21.4%	19.2 ± 8.0	23.3 ± 9.4	26.6 ± 18.7	1.3	1.4
TR85	533.9 ± 207.7	624.0 ± 170.9	17.1% ^∗^	16.9 ± 7.5	19.9 ± 9.3	18.3 ± 17.2 ^∗^	672.1 ± 228.3	762.90 ± 258.98	13.5% ^∗^	21.3 ± 8.4	24.3 ± 9.8	14.4 ± 13.3 ^∗^	1.3	1.3

*^∗^Significant difference to CON at *p* = 0.05.*

### Posture

A classification of the test persons based on the average *flèche lombaire* percentage of 8.06% (see Table 2) shows that all test persons together constitute a group with poor posture. Proprietary, unpublished data of 724 test persons showed a *flèche lombaire* percentage of 6.92% ± 3.06 (95% confidence interval 6.63–7.21%) for a normally pronounced lumbar lordosis and a mean value of 9.17% ± 3.59 (95% confidence interval 8.76–9.58%) for a hyperlordosis. Accordingly, we had expected that all our test persons would notably benefit from a strengthening of the posture-straightening muscle groups. However, changes in posture parameters were identified only for trunk anteversion, which improved for all groups including the control group. We think that this improvement was caused by a learning process, more specifically with an increased familiarity with the testing procedure, because upper body anteversion is easy to consciously improve by activating the dorsal muscle chain. We assume that the test persons unconsciously straightened their body posture due to the familiarity with the testing situation during the post-tests. In contrast to the sagittal tilt of the body, the depth of the lordosis of the cervical and the thoracic spine are more difficult to change deliberately and require an excellent body awareness (Singla and Veqar, 2017). The WB-EMS training activated the muscle groups of the trunk, the pelvis and the legs unspecifically. This means that the simultaneous stimulation by large surface electrodes activated all muscle fibers underneath. Agonists and antagonists were simultaneously activated and, depending on the exercise, the muscles actively involved in the movement were additionally activated by the central nervous system. Therefore, it is plausible that both the dorsal and the ventral muscle chains were exercised to the same extent. Looking at the isometric strength ratio of extension and flexion (extension/flexion quotient), the values found in this study correspond to the values determined in other studies (Smith et al., 1985; Beimborn and Morrissey, 1988; Kim et al., 2006). For the isometric strength and torque ratios among the groups, no significant differences were determined, even if the absolute values in some instances significantly increased (Table 3).

The reduction of a pronounced lumbar lordosis is considered an important therapeutic approach because interrelationships with increased degeneration are known, in particular in the facet joints (articulationes processuum articularium) (Murray et al., 2017). It is also known that the position of the pelvis in the sagittal plane plays a key role in the reduction of lumbar lordosis (*flèche lombaire*) (Buchtelová et al., 2013). Analyses of adolescents with poor posture came to the conclusion that targeted training of the pelvis-straightening musculature (*M. rectus abdominis*, *M. gluteus maximus*, *M. biceps femoris*, *M. semitendinosus*) improved the habitual position of the pelvis (Ludwig et al., 2016). We suspect that the simultaneous training of the pelvis-straightening muscles (see above) and the pelvis-flexing muscles (*M. erector spinae lumbalis*, *M. quadriceps femoris*) counterbalanced the influence of both muscle groups on the pelvis position. In addition, the correction of pelvis or spinal column curvatures works best when the test persons exercise their body perception (Bansal et al., 2014; Ludwig et al., 2016). This, however, was not part of the WB-EMS training. Paillard emphasizes that EMS is not able to improve the coordination between agonist and antagonist muscles and therefore does not support the coordination of complex movements (Paillard, 2008). Even if the training did strengthen the muscles, the awareness of how to utilize specific muscle groups for posture correction was apparently not improved. However, this awareness seems to be a key factor in conscious posture correction (Woollacott and Shumway-Cook, 1990; Ludwig et al., 2016).

We consider this a limitation of the WB-EMS training because this shows that merely (and demonstrably) strengthening the muscles does not have any direct effect on processes controlled by the central nervous system, such as body posture. The interaction of sensory information and motor activity in the form of a regulation process (Feldman et al., 2014; Chiba et al., 2016) can probably not be improved easily. Even if movements were actively performed during stimulation, it remains unclear which central-nervous learning processes run when conscious muscle activity overlaps with externally triggered muscle activity. Some studies even suspect a negative effect of EMS training on central-nervous learning processes in this context (Paillard, 2012).

Although we did not determine any improvement of body posture through WB-EMS training, we need to point out that a decline was not determined, either. Possible reasons that might have caused an increase in the forward tilt of the pelvis by changing the muscular balance of the muscle groups connected to the pelvis have already been discussed. To sum it up, we can say that while unspecific training was able to result in a strength increase of the trunk muscles, it did not have any direct effect on habitual posture.

### Limitations

Our study has some limitations. Firstly, we tested healthy test persons only. On average, the test persons exhibited a relative lumbar lordosis depth of 8°, meaning they were close to poor posture, but symptom-free. Therefore, the results cannot be transferred directly to patients with extensive poor posture and low back pain. Secondly, the WB-EMS training was unspecific. This means that the specific muscle groups required for posture corrections were not trained exclusively and selectively. However, we deliberately decided to apply an exercise program as it is preset by device manufacturers and used in many gyms. We must also note that the forces of trunk extension and flexion were measured only summarily. We were not able to measure the forces of other, possibly relevant muscle groups (e.g., gluteal muscles, thigh muscles, neck muscles). However, these muscle groups may influence the position of the pelvis and thus, under certain circumstances, the *flèche lombaire* or the position of the thoracic spine and thus the *flèche cervicale*. Furthermore, our test persons were all inexperienced in WB-EMS training and not actively involved in sports. Even though we see this as one of the strengths of our study, we cannot transfer the results directly to athletes.

## Conclusion

A 10 weeks whole-body electrostimulation training with a stimulation frequency of 85 Hz in contrast to training with a stimulation frequency of 20 Hz improves the trunk muscle strength of an untrained group but does not significantly change posture parameters.

## Ethics Statement

This study was carried out in accordance with the recommendations of the ethics commission of Fachbereich Sozialwissenschaften, Technische Universität Kaiserslautern, Germany with written informed consent from all subjects. All subjects gave written informed consent in accordance with the Declaration of Helsinki. The protocol was approved by the ethics commission of Fachbereich Sozialwissenschaften, Technische Universität Kaiserslautern, Germany under No. 02/17.

## Author Contributions

OL was involved in the design and implementation of the study, and wrote the manuscript. JB was involved in the design and implementation of the study, and helped in the writing of the manuscript. SB was involved in the implementation of the study. WK and MF was involved in the design of the study and helped in the writing of the manuscript.

## Conflict of Interest Statement

The authors declare that the research was conducted in the absence of any commercial or financial relationships that could be construed as a potential conflict of interest.
